# Cardiovascular Disease Prevention in Adolescents: eHealth, Co-Creation, and Advocacy

**DOI:** 10.3390/medsci7020034

**Published:** 2019-02-24

**Authors:** Rebecca Raeside, Stephanie R. Partridge, Anna Singleton, Julie Redfern

**Affiliations:** 1 Westmead Applied Research Centre, Faculty of Medicine and Health, The University of Sydney, Westmead, NSW 2145, Australia; stephanie.partridge@sydney.edu.au (S.R.P.); anna.singleton@sydney.edu.au (A.S.); julie.redfern@sydney.edu.au (J.R.); 2 Prevention Research Collaboration, Charles Perkins Centre, Sydney School of Public Health, Faculty of Medicine and Health, The University of Sydney, Camperdown, NSW 2006, Australia; 3 The George Institute for Global Health, The University of New South Wales, Camperdown, NSW 2006, Australia

**Keywords:** adolescent, cardiovascular disease, co-creation, eHealth, advocacy, risk factors

## Abstract

Cardiovascular disease (CVD) is the leading cause of death globally. Early atherosclerotic changes can begin to occur early in life and though adolescence. The prevalence of modifiable CVD risk factors, namely, smoking, poor diet quality, excessive alcohol intake, physical inactivity, and overweight and obesity can exacerbate the early onset of atherosclerosis. There is a need to improve modifiable risk factors during adolescence to prevent progression to CVD in later life. Electronic health (eHealth) behaviour change interventions are a potential solution for adolescents to improve CVD risk factors, given adolescents are digital frontrunners and digital technology is wide-reaching. The process of co-creating eHealth behaviour change interventions with adolescents is a promising strategy to improve intervention effectiveness and engagement. Additionally, effective youth advocacy is an emerging strategy for CVD prevention in adolescents. This narrative review evaluates published eHealth behaviour change interventions targeting cardiovascular disease risk factors in adolescents, which utilize a co-creation process, describe the emerging role of advocacy in CVD prevention for adolescents and provide recommendations for future interventions.

## 1. Introduction

Cardiovascular disease (CVD) is one of the most significant public health challenges facing today’s adolescents, aged 10–24 years [1,2]. Adolescents now account for over 25% of the global population—the single largest population segment in history [3]. For the past 15 years, coronary heart disease and stroke have remained the leading cause of mortality and morbidity, globally [4]. Currently, 1.1 billion people have hypertension and 17.9 million people die each year from CVD—31% of all global deaths [2]. In developed countries and regions, such as Australia, USA, and Europe, 29%, 24%, and 45% of all deaths are from CVD, respectively [5,6,7]. However, over 75% of all CVD deaths occur in low- and middle-income countries [8]. If current trends persist, experts predict over 23 million people will die from CVD in 2030 [2]. Although clinical signs of CVD usually present in adulthood, early atherosclerotic changes are occurring during adolescence [9,10] and may be mediated by modifiable CVD risk factors [10,11,12]. Moreover, the existence of modifiable CVD risk factors, namely, smoking, alcohol intake, diet quality, physical activity, and overweight and obesity [13], during adolescence increases the chances of having a poor cardiovascular risk profile in adulthood [13,14,15]. Therefore, the prevention of CVD in adolescents is of the utmost importance for researchers, health professionals, and policymakers to prevent premature death from CVD in future generations. 

With the increasing population of adolescents and the growing demand for prevention services, traditional in-person services are insufficient alone to meet demand. Innovative and contemporary population-based solutions are needed for the adoption of healthy lifestyle risk factors to prevent CVD in adolescents. Adolescents have been born into the digital age and are considered digital frontrunners. The global reach and integration of digital technologies in the lives of today’s adolescents have the potential to deliver CVD prevention interventions *en masse*. Such interventions, namely, electronic health (eHealth) allow consumers to obtain health services online from global providers [16]. Researchers and health professionals can deliver eHealth interventions via computers, tablets, smartphones, and wearable devices, such as smartwatches or activity trackers [16,17]. Interventions using eHealth have gained popularity in recent years due to the assumption that such interventions remove barriers of traditional in-person services, including accessibility and engagement. In fact, between 2000 and 2016, researchers published 1716 studies utilizing eHealth for physical activity, sedentary behaviour, and diet lifestyle interventions [18]. However, efficacy or effectiveness of eHealth interventions is reliant on successful engagement, which is complex and provides significant challenges [19,20].

Adolescents are digital frontrunners, early adopters and they often have unique healthcare preferences. Yet, adolescents are a difficult group to target, as they are generally in good health and do not visit health professionals regularly. Therefore, eHealth interventions tailored for adolescent populations are a potential approach to overcome the issue of accessibility. A key strategy to increase adolescent’s engagement with preventative eHealth services is to engage them in the co-creation process. Youth advocacy also has the potential to be a powerful and effective strategy for the prevention of CVD in adolescents. Through co-creation and advocacy, researchers and health professionals can potentially optimize the quality of the health promotion services offered to young people and, ultimately, help prevent the early onset of CVD in youth. Adolescents may become involved in co-creation and advocacy through school, university, sports, religious, social, or community groups. However, the ways that adolescents can become involved is certainly not limited to constructed groups. When considering youth advocacy, adolescents often become involved pro-actively due to personal interest and, in a digital era, adolescents can potentially be engaged using social media, through targeted advertising, and via online groups. Considering this, this narrative review will now examine co-creation and youth advocacy as strategies to increase engagement with and effectiveness of eHealth interventions to improve CVD risk factors in adolescents. This is not intended to be an exhaustive compilation of every individual study, however, a descriptive narrative review conceptualizing both co-creation and eHealth in the context of CVD prevention for adolescents using contemporary examples.

## 2. What is Co-Creation?

Researchers developed the co-creation process from participatory design [21,22]. Experts agree that successful interventions are often co-created with the end-user [23]. In research and preventative healthcare, co-creation involves engaging with all stakeholders (users) throughout the entire design and development process [24], to ensure the intervention meets their needs and is usable. Co-creation involves more than users saying what they would like in an intervention [25]. Instead, they are actively involved in each phase of the design and development process, by exploring their behaviours and needs and being actively engaged in developing solutions [26]. For researchers and health professionals, the co-creation process allows for the development of tailored behavioural interventions for the end-user, which means that the intervention is more likely to be engaging, satisfying, and appropriate [27].

The first stage of co-creation is to engage with the end-user and to understand their current behaviours, needs, and facilitators necessary for behaviour change [21]. When developing behavioural interventions, the end goal for researchers and health professionals is to affect behaviour change [26]. Intervention designers can increase the chances of achieving behaviour change, by designing the intervention with the end user, as it provides a different perspective and consumer insights. The sessions of co-creation are essential to establish collaborative relationships. Collaborative sessions will ensure that the participants are engaged and feel that they are in a comfortable environment to explore their lived experience. Moreover, participants will be more likely to ascertain their opinion of whether an intervention-style will work or not [28]. 

Co-creation is most effective when it is an iterative process, meaning the researchers or health professions work with a representative of the end user to design the intervention, and then test it in a pilot setting [25,26]. After pilot testing, it is essential to evaluate and refine the design for future testing. This process allows for the development of the best possible intervention before implementation.

## 3. Co-Creation, Adolescents, and eHealth

Adolescents live in an ever-changing environment where digital technologies are developed and changed so rapidly that it is a challenge to keep up to date [29]. One strategy to increase engagement with digital technologies for health-related behaviour change is the utilization of co-creation [30]. Adolescents are a crucial group to involve in co-creation of eHealth behaviour change interventions due to their understanding of digital technologies and constant willingness to adapt to the ever-changing digital technology landscape [31]. Currently, adolescents have low uptake of health interventions across many areas [32,33,34], due to a lack of initial engagement. Researchers hypothesize that by co-creating health interventions with adolescents, adolescents and their peers will be more likely to actively engage with the intervention, which will be useful in the long-term [28]. 

Co-creation sessions should allow for a shared understanding and shared language between the participants and researchers [35]. Therefore, it is essential to choose appropriate methodologies when involving adolescents in co-creation sessions. First, researchers should consider hosting short sessions and taking appropriate breaks to keep adolescents engaged. Second, before the session, researchers should decide on the most appropriate content and style for a young audience. A co-creation session will be most effective if the adolescents are entirely engaged and feel comfortable to share their experiences. Therefore, co-creation sessions should be fun and enjoyable, as well as productive in gaining trust and developing a platform for sharing experiences. Additionally, to investigate ideas further, it is essential researchers have a semi-structured session with a set of overarching ideas to explore and be flexible to allow the exploration of relevant new ideas [28].

Co-creating eHealth interventions does bring another layer of complexity as the technology developers of interventions also need to be involved in the co-creation process to ensure that the participants ideas are practical and technologically possible [28]. For example, if the participants wanted certain features of a smartphone application (app), but the app developers could not make it happen, it is vital for the participants and the app developers to work together to find a possible solution. Therefore, iterative design is essential so that the developed intervention can be tested and refined, based on the user’s needs.

## 4. Co-Creation of eHealth Interventions for the Prevention of Cardiovascular Disease in Adolescents

This section identifies and discusses eHealth lifestyle interventions targeting one or more CVD risk factors, which have been co-created with adolescents. After searching PubMed, Medline, and grey literature using the search terms ‘adolescents’, ‘co-creation’, ‘eHealth’, and ‘CVD prevention,’ six studies were identified as examples that involved adolescents (10–24 years), co-creation, eHealth, and CVD prevention. One study targeted risky single-occasion alcoholic drinking, one targeted lifestyle intervention, and four targeted weight management interventions. Table 1 presents a summary of the eHealth interventions. 

### 4.1. Mobile Intervention for Drinking in Young People

Mobile intervention for drinking in young people (MIDY) is a co-created mobile phone survey sent to adolescent participants, aged 18–25 years, at hourly intervals on a night out and the next day to monitor drinking events [36]. The co-creation of this intervention took place through development workshops consisting of three components: focus group discussions, media analyses, and a design session. Following the workshops, researchers developed the survey and intervention content incorporating new ideas that had been generated through the workshops to ensure the survey was feasible and acceptable to participants. The researchers and workshop participants collectively decided that the best way to complete the intervention was to send hourly messages from 7 p.m. to 2 a.m. Feedback was provided to the participant when they responded to the initial text message. A follow-up text message was also sent at 12 p.m. the next day. Through co-creation, the researchers found that hourly messages would be socially acceptable and feel familiar, like texting a friend. They also found that messaging felt non-judgmental, which may encourage participation, as participants reported they were unconcerned with their privacy in reporting drinking and adverse events because of drinking [42]. The co-creation of the MIDY intervention with adolescents led to the inclusion of broader contextual information, a more personalized feel and minimal intrusion into their planned night. The researchers proceeded to conduct a randomized controlled trial (RCT) using the text message intervention but found that that there was no significant difference in risky single-occasion drinking in the intervention groups [43].

### 4.2. Wearable Sensors for Adolescents Who Are Overweight

Another example identified from the literature was a study outlining the co-creation of garments with wearable sensors, with adolescents aged 13–17 years and the testing of the garments [37]. Co-creation took place through focus groups which explored the adolescents’ opinion of health, the importance of a healthy and active lifestyle, the use of mobile and social media for health and the importance of monitoring their health through wearable devices. Co-creation occurred through three phases: phase one included exploring the level of adolescents’ awareness of technology for health, phase two explored the use of sensors and wearable devices for health management, and phase three was focusing on the co-creation of a wearable device suitable for adolescents. The researchers found that adolescents were interested in wearable sensors and devices and the associated data that they detect, but devices must be comfortable and practical. The researchers concluded that co-creation was an effective and reliable method in providing solutions for the adolescents’ requirements and preferences, which increased daily use of the designed products.

### 4.3. Cell Phone Intervention for You Trial

The Cell phone Intervention for You (CITY) Trial was an RCT comparing two active weight management interventions to a usual care control group among overweight and obese adolescents and young adults aged 18–35 [38]. The researchers delivered one of the two active interventions through an automated mobile phone application. The first intervention group received personal coaching along with smartphone self-monitoring and the second intervention group received the interactive mobile application via cell phone. The researchers developed the mobile phone application during the first year, or developmental phase, which encompassed focus groups and iterative, participatory design with adolescents and young adults. Focus groups were used to generate ideas including advertising strategies, message framing, advertising avenues, application format, and content. Lin et al. identified the co-creation process as a unique opportunity to interact with participants. Through the co-creation process, participants identified their preferences for intervention tailoring, i.e., individually-targeted behaviour change through personalized goal setting. Despite the formative co-creation phase, the authors identified three main challenges during the RCT: (i) the fast-changing nature of mobile technology; (ii) maintaining the collaboration between the designers of the lifestyle intervention and the mobile technology experts who are required for correct intervention delivery; and (iii) maintaining engagement with the participants. Over 24 months, researchers found that the group who used the co-created mobile app were not superior to the control group in any measurement. The personal coaching group had significant weight loss at six months, but not at 12 or 24 months [44]. 

### 4.4. Lifestyle Intervention for Rural Young Adult Overweight and Obese Males

The Fit for Young Adult Males (Fit4YAMS) text message-based lifestyle intervention was developed using focus group discussions with rural adolescent and young adult males, aged 18–25 years [39]. The focus groups were semi-structured and explored the young adult males’ preferences for structure and intervention delivery. During the co-creation process, Bailey et al., found that rural young adult males preferred a personalized intervention. Personalized or tailored interventions require additional resources and costs to deliver. However, if the intervention is personalized, the researchers hypothesized the intervention might be more effective and cost-effective. For the personalized intervention, each participant would go through a scheduled personalization session where certain questions would be asked to ascertain their schedule, likes and dislikes, and goals. The researchers also discussed text message frequency in the focus groups. The participants agreed that the frequency of text messages was important—too many text messages would feel like nagging and too little would likely have no effect. Additionally, researchers and young adult males discussed collecting feedback throughout the intervention to inform adjustments to the program, which would allow for maximum engagement.

### 4.5. Computer-Based Weight Management Program Using Avatars and Virtual Coaches

LeRouge and colleagues concentrated on the potential capability of avatars (representing the user) and virtual agents (representing the coach) to deliver computer-based weight management interventions for adolescents (age not specified) [40]. Self-management of weight can be a complex journey for adolescents, who must balance multiple behavioural changes, including nutrition, exercise, motivation, and socialization. Therefore, LeRouge and colleagues hypothesized that using avatars may assist adolescents to overcome some of the barriers. The development of the lifestyle intervention utilized co-creation in two phases: phase one included focus groups with adolescents, parents and health care providers and phase two was a prototype assessment by adolescents and healthcare providers. Through co-creation, the researchers discovered that the use of avatars can increase access and extend the reach of existing medical programs, as well as provide motivating experiences for adolescents and increase engagement. Avatars can also be altered in appearance and language and, therefore, reach adolescents of different ethnic and language backgrounds. Avatars who are delivering health interventions are perceived as non-judgmental by adolescents. As a result, adolescents may be more honest and revealing to an avatar than they would be in a face-to-face encounter with a healthcare professional, but further research is needed to explore this further. 

### 4.6. Mobile App Design for Weight Management in Adolescents with Complex Needs

Rivera and colleagues engaged adolescents, parents and health care providers in the design and development of a mobile phone app for weight management in adolescents, aged 12–18 years who have complex needs [41]. To co-create the mobile phone application, researchers used separate semi-structured focus groups with each group of stakeholders. The researchers analysed the data collected via the focus groups and found that the app features should enhance existing weight management, improve long term maintenance of healthy behaviours and the app development should be through an interactive process. The stakeholders identified key features necessary in the app, including support for healthy eating, mental health, social support, self-monitoring, communication with healthcare providers, and gamification or incentives. Through the focus groups, the researchers also identified that long-term tracking within the app would support adolescents to make healthier choices, which would benefit their long-term health. The researchers hypothesized the app would enhance healthcare access for adolescents with complex weight management needs and provide a continuity of care between face-to-face visits with healthcare providers. Moreover, the app may enhance the already established healthcare provider and patient relationship and provide more personalized and meaningful support. 

## 5. What Is Youth Advocacy?

Experts and international health organizations define advocacy as the process by which individuals or groups increase public support for, or recommendation of, a cause or policy at a community, state, national, or international level [45]. Advocacy is an action-oriented process that involves providing solutions to decision makers, who have the power to implement change [46]. Advocacy usually involves political systems, the media, communities, and organizations. The focus of advocacy for CVD prevention is often about seeking the support of decision-makers to gain policy and systems support for environment changes, new or updated policies, or implementation of public health programs. Advocacy is not a new concept and the World Health Organization has recognized advocacy as one of three primary strategies for achieving health promotional goals since 1986 [47]. Advocacy is most effective when it involves a representative sample of society [48,49]. However, youth voices are often unheard in advocacy efforts, despite CVD being one of the greatest public health challenges for the current generation of young people. Additionally, youth advocacy has many benefits, including education, skill development, and behaviour and attitude changes [48]. 

Advocacy has the potential to be a powerful and effective method for the prevention of CVD in adolescents [50]. Firstly, inaction on the issue will result in young people being profoundly affected by CVD in the long-term [51]. Our society has entrenched unhealthy behaviours and environments, and failure to address such issues will ensure CVD continues to affect future generations. The earlier onset of CVD for young people, including poor health, financial burdens, and reduced quality of life, may motivate adolescents to act [13]. Secondly, young people naturally form groups, which in turn may generate greater change and impact on CVD prevention. When young people come together to form groups, everyone has inputs that have the potential to influence their own and each other’s behaviours and ways of thinking. Inputs may include their knowledge and experiences, their social environment, built environment, or political environment [48]. The accumulation of everyone’s inputs gives them the knowledge and enthusiasm to advocate for real change to their community and beyond. A potential reason that youth may have the ability to maximize change and impact is because of their age and, in some sense, their naivety. Decision-makers may be less questioning about their motives and less likely to reject their requests [52]. In this way, young people hold a powerful position in being able to effect change. Finally, the benefits of youth advocacy can be bi-directional, with not only societal changes but also changes within the individual. Bi-directional means the advocacy effects that are positive to society may also be positive to the individual. When young people are involved in advocacy movements, it may increase their feelings of empowerment, increase self-esteem and self-efficacy [53,54], and also increase their knowledge and skills in the prevention of CVD. In turn, advocacy may motivate adolescents to reduce their risk of CVD through the knowledge that they gain as being part of a larger advocacy movement. 

## 6. Youth Advocacy Strategies for the Prevention of Cardiovascular Disease in Adolescents

For the prevention of CVD in adolescents to be on the forefront of the global agenda, there needs to be a dramatic shift in current advocacy strategies. Advocacy strategies for adolescents can include: (i) sharing targeted messages for preventive health via new media channels, including social media, (ii) co-creation of health promotion programs; (iii) leading health promotion programs to promote healthier behaviours to their peers; or (iv) engaging with local, national or international youth advisory groups, organizations or social movements to advocate for policies that aim to improve CVD prevention. Table 2 presents a summary of the youth advocacy strategies.

## 7. Future Directions for Adolescent Engagement through Co-Creation, eHealth, and Youth Advocacy

**Figure 1:**
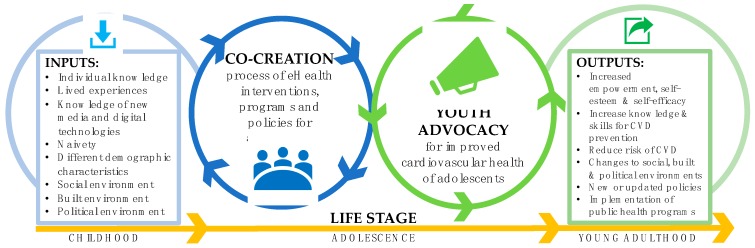
A conceptual model of the inputs and outputs of the co-creation and youth advocacy processes for cardiovascular prevention during adolescence.

There is an urgent need to engage adolescents in planning and decision-making processes that will affect their cardiovascular health now and in the future. Adolescents can raise awareness and enable change in the CVD landscape. As outlined in this review, strategies that researchers and health professionals can use to engage adolescents in the prevention of CVD, including employing co-creation methodologies when developing eHealth interventions and enabling and fostering avenues for youth advocacy. Figure 1 presents a conceptual model of these strategies.

Co-creation is a potentially useful way to increase engagement with eHealth interventions. This narrative review discussed six studies, however, it is likely that there may be more published studies. In the studies discussed, adolescents became involved in the co-creation process through a vast number of methods, including through schools, summer camp, print media, social media, hospital settings, and even face-to-face approaches [36,37,38,39,40,41]. The variety of recruitment methods between studies demonstrates the complexities in recruiting adolescents for co-creation of health interventions, and health interventions more broadly. In addition, there are currently no best practice approaches for the recruitment and engagement of adolescents to health interventions and further research is required to identify the most effective approaches. Researchers should also carefully consider the structure and design of co-creation sessions and process to maximize participation and ensure the exploration of all aspects of the adolescents’ social, built, and political environment. Most eHealth interventions discussed in our review consisted of semi-structured focus group sessions during the formative phase of the intervention design, which may not be flexible enough to allow for the full development of ideas, concepts, and adaptations, particularly during critical periods, such as when the intervention is being implemented. Moreover, utilizing other activities, such as media analyses, in addition to workshops or focus groups, may be an effective strategy to increase adolescents’ engagement and willingness to engage and share their honest opinions. To maintain engagement over time, researchers can consider an adaptive intervention design. This design allows for continued adolescent engagement in the entire research process and allow for the intervention to be altered based on feedback from adolescents at given time points to ensure maximal engagement. Further research should aim to employ such designs to increase the engagement of adolescents in design and implementation of eHealth interventions. 

Youth advocacy is another avenue to explore for effectively engaging adolescents in eHealth interventions. Though, adequate training must be provided to youths to ensure they know how to be effective advocates. Additionally, it is essential that adult leaders of youth advocacy groups are encouraging and empowering. Advocacy and co-creation are complex processes. Currently, there are no clear frameworks or models to guide researchers or health professionals about how to incorporate such processes into their intervention development. Future research should focus on developing frameworks or models. In Figure 1, we demonstrate the potential benefits from incorporating the two concepts into CVD prevention for adolescents. The potential health and social benefits for adolescents of co-creating and advocating for CVD prevention interventions have the potential to flow from adolescence into adulthood. Utilizing these two processes when designing eHealth interventions may assist researchers to ensure their interventions are both effective and engaging for adolescents. 

## 8. Conclusions

Due to the existence of modifiable CVD risk factors in adolescents increasing the chances of a poor CVD risk profile in adulthood, and the increasing adolescent population, adolescents are a key group to target for CVD prevention. Strategies used to increase engagement and effectiveness are not limited to those discussed herein. Co-creation, youth advocacy, and eHealth are a few of many approaches to employ in the battle against CVD. Given the potential synergistic effects of co-creation, youth advocacy, and eHealth for prevention of CVD in adolescents, they are promising avenues for future research efforts. Future research should aim to test the effects of co-creation and youth advocacy for eHealth interventions to increase the evidence-based strategies to improve adolescent engagement in CVD prevention interventions and their effectiveness. 

## Figures and Tables

**Table 1:** Summary of eHealth lifestyle interventions targeting one or more CVD risk factors that have been co-created with adolescents.

Study Details				Co-Creation Strategies		
First Author, Year	Study Name	Country	Population	Stakeholder(s)	Methodology	Outcomes
Wright et al. 2016 [36]	MIDY	Australia	18–25 years	Adolescents and young adults	Workshops	The intervention was considered feasible and acceptable
Standoli et al. 2016 [37]	Wearable sensors	Italy, Spain, and the UK	13–17 years	Adolescents	Focus group discussions	Effective and reliable solution to match users’ requirements and preferences
Lin et al. 2015 [38]	CITY Trial	USA	18–35 years	Adolescents and young adults	Focus group discussions	Unique opportunity to interact with participants, participants identified a preference for intervention tailoring
Bailey et al. 2018 [39]	Fit4YAMS	Australia	18–25 years	Adolescents and young adults	Focus group discussions	High degree of personalization needed for optimum engagement
LeRouge et al. 2016 [40]	Avatars and virtual coaches	USA	Not specified	Adolescents, parents and healthcare providers	Focus group discussions	Increased probability of engagement and long-term retention of users
Rivera et al. 2018 [41]	Mobile app design for teens	Canada	12–18 years	Adolescents, parents and healthcare providers	Focus group discussions	Identified necessary features of the app and that the interface should be simple and interactive

eHealth: congenital vascular disease; CVD: congenital vascular disease; MIDY: mobile intervention for drinking in young people; CITY: cell phone intervention of you; Fit4YAMS: fit for young adult males.

**Table 2:** Summary of youth advocacy strategies and examples from studies targeting one or more CVD risk factors.

Health Promotion Advocacy Strategy		Brief Description	Potential Reasons for Effectiveness	Example Study	Advocacy and/or Behavioural Outcomes
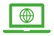	Targeted messages via social media	Allows adolescents to create and share content within their social networks and beyond	Personally relevant content Potential to evoke strong, positive emotional responses Likely to be shared widely amongst new audiences	SaludABLEOmaha ^1^ [55]	↑ community in readiness for change from ‘vague awareness’ to ‘preparation’
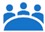	Co-creation of programs	Active process of engagement which creates awareness about CVD prevention and adolescents are directly involved in solution generation	Increase feelings of empowerment and self-efficacy about CVD prevention	Youth Engagement and Action for Health! ^2^ (YEAH!) [56,57]	↑ self-efficacy and participation in advocacy ↑ assertiveness, health advocacy history, knowledge of resources, social support for health behaviours ↑ physical activity levels
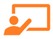	Peer-led programs	Adolescents leading health promotion programs for their peers	Peers have a greater influence on health behaviours of than parents, teachers or health professionals Improvements in peer leaders lifestyle behaviours	Students As LifeStyle Activists (SALSA) ^3^ [58]	↑ fruit & vegetable intake ↓ sugary drinks ↑ mod-vig physical activity (boys only) ↑ daily breakfast intake (boys only)
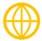	Youth advisory groups, organizations or social movements	Connects young people who have a shared passion or interest for CVD prevention	Connects individuals locally, nationally and internationally Creates a shared voice on CVD prevention	The North Carolina (NC) Youth Empowerment Study (NCYES) ^4^ [59]	↑ number of youth groups in NC focused on tobacco use prevention & control ↑ positive change in school tobacco use policies in several school districts

^1^ SaludABLEOmaha Initiative utilising youth advocacy and social media to increase readiness of residents in a Midwestern Latino community to address obesity and adopt healthy lifestyles. ^2^ The YEAH program was designed to engage youth and adult group leaders in projects that could positively impact on nutrition and physical activity environments in their school and neighbourhood. ^3^ The SALSA program is a peer-led school-based intervention for the prevention of cardiovascular disease, which implemented an educational program designed to motivate secondary students to increase physical activity, decrease recreational screen time and improve food and beverage choices. ^4^ The NCYES is a study that includes the establishment of a youth advisory board for tobacco use prevention activities. ↑ increase; ↓ decrease; !
